# Solar prominences: theory and models: Fleshing out the magnetic skeleton

**DOI:** 10.1007/s41116-018-0016-2

**Published:** 2018-10-22

**Authors:** Sarah E. Gibson

**Affiliations:** National Center for Atmospheric Research, 3080 Center Green Dr., Boulder, USA

**Keywords:** Solar prominences, Magnetohydrodynamics, Corona: structures, Prominences: Magnetic fields, Prominences: Models

## Abstract

Magnetic fields suspend the relatively cool material of solar prominences in an otherwise hot corona. A comprehensive understanding of solar prominences ultimately requires complex and dynamic models, constrained and validated by observations spanning the solar atmosphere. We obtain the core of this understanding from observations that give us information about the structure of the “magnetic skeleton” that supports and surrounds the prominence. Energetically-sophisticated magnetohydrodynamic simulations then add flesh and blood to the skeleton, demonstrating how a thermally varying plasma may pulse through to form the prominence, and how the plasma and magnetic fields dynamically interact.

## Introduction

**Fig. 1:**
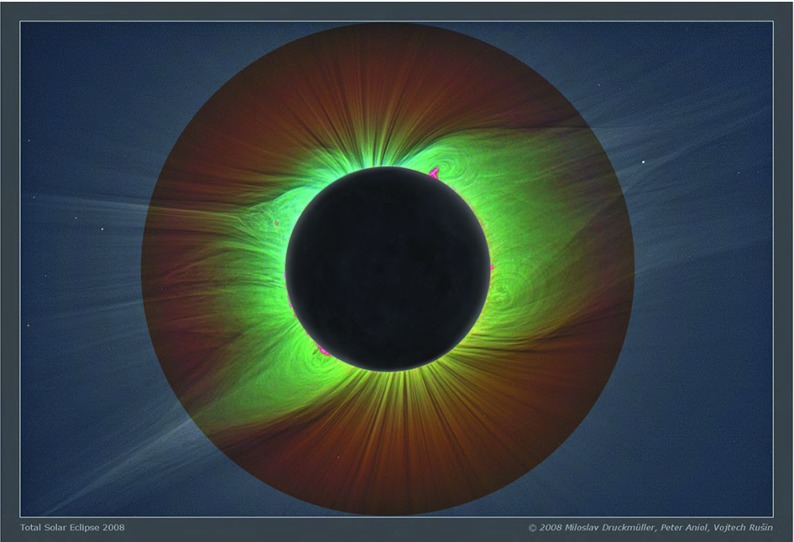
Prominences in true color during 2008 total solar eclipse. Image reproduced with permission from http://www.zam.fme.vutbr.cz/~druck/eclipse/, copyright by Miloslav Druckmüller, Peter Aniol, Vojtech Rušin

Prominences are surprising. During a solar eclipse they are visible as dischordantly clumpy and bright (indeed, pink,) structures, sharply contrasting with the elegant pearly white streamers that make up most of the corona (Fig. 1). As we learn more about prominences through multiwavelength, high-resolution observations, the mysteries surrounding them only intensify. How can something that is relatively cool and dense be suspended in a hot and sparse atmosphere? Where do they come from, and how do they go?

The answers to these questions clearly involve magnetism, the controlling force of the solar corona. In particular, the abiding presence of a prominence implies a magnetic structure that can provide sustained gravitational support and thermal isolation. Several excellent review chapters within the recent book *Solar Prominences* (Engvold and Vial 2015) cover our theoretical understanding of the origins of such magnetic structures and their evolution on a variety of temporal and spatial scales (Mackay 2015), the energetic processes associated with prominence formation and visibility (Gilbert 2015; Heinzel 2015; Karpen 2015), and the mechanisms responsible for the ultimate fate of many prominences as the cores of solar eruptions (Fan 2015). Prominence observations and interpretation are also well-covered by other chapters within that book (Ballester 2015; Engvold 2015; Gibson 2015; Gopalswamy 2015; Kucera 2015; Labrosse 2015; López Ariste 2015; Lugaz 2015; Martin 2015; Parenti 2015; Webb 2015) and by the *Living Reviews* on “Prominence Oscillations” by Arregui et al. (2018), and “Solar Prominences: Observations” by Parenti (2014). Additional comprehensive reviews of prominence structure, dynamics, and physical processes are found in Labrosse et al. (2010) and Mackay et al. (2010). Finally, for a big-picture context to prominence theory and models, we refer the reader to Low (2018), a recent review on “Coronal Magnetism”.

The focus for this review is the physical state of the non-erupting prominence, i.e., the nature of its magnetohydrodynamic (MHD) quasi-equilibrium. In particular, in Sects. 2 and 3, we review models of the magnetic structure—or skeleton—of the prominence and its coronal environment. In Sect. 4, we consider prominence dynamics and thermodynamics, and present a brief overview of the state of the art in numerical simulations, where MHD is coupled to energy transport. Finally, in Sect. 5 we reflect upon some general conclusions and expectations for the future.

## The prominence magnetic skeleton

Prominences are in many ways heterogeneous. They vary spatially in size and location, and vary temporally in evolution and dynamics. This complicates statistical analyses and classification schemes (Tang 1987; Mackay et al. 2008; Engvold 2015). Even so, many commonalities are observed (Parenti 2014). Over the past several decades, these have been used to build physical models of the prominence.

The generality and applicability of these models can be explored by comparing to a variety of observations, and the models themselves made more “realistic” through the use of complex magnetic boundaries. It is important to bear in mind, however, that—as we will discuss in Sect. 4—prominence dynamics and thermodynamics challenge many of the assumptions of these relatively simple models. Nevertheless, such models provide a framework for understanding the prominence as a fundamental magnetic structure of the solar corona.

### Prominence bones: magnetic dips

The central portions, or “spines”, of prominences are generally observed to be localized to a vertical sheet suspended above and at an angle of about $20^\circ$–$35^\circ$ to an underlying magnetic polarity inversion line (PIL; Parenti 2014). Early observations of prominence magnetic fields found predominantly horizontal magnetic fields within this vertical sheet of a magnitude implying that magnetic pressure dominates over the thermal pressure in the prominence (low plasma $\beta$), and with this magnitude increasing with height (Rust 1967; Leroy 1977, 1978) (see Sect. 4.2.2 for further discussion). Since a low-$\beta$ plasma equilibrium is one in which magnetic forces balance (“force-free” fields), these observations taken together imply a downwards magnetic pressure gradient force which in turn requires an upwards magnetic tension force. These characteristics, along with the observed longevity and general stability of prominences, led to a fundamental building block for early prominence models, namely, the requirement that the cool prominence mass be supported within dipped magnetic fields. (Note that we will revisit the absolute necessity for this requirement in Sect. 4.2.1.)

**Fig. 2:**
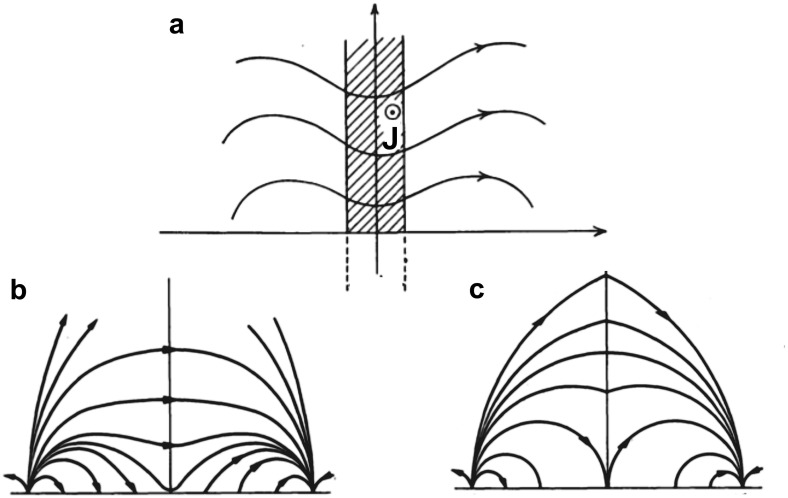
Cross sections of 2.5D arcade-type prominence models. **a** Summary representation of Kippenhahn and Schlüter concept (note, current is pointing *out of* the page.) Image reproduced with permission from Anzer (1969), copyright by D. Reidel. **b** Example of analytic quadrupolar potential configuration with dips suitable for the formation of a stable prominence, that **c** may evolve to a non-potential configuration where prominence mass is supported by forces associated with a current sheet at the center of the dips. Image reproduced with permission from Kippenhahn and Schlüter (1957), copyright by Springer

### Early prominence models

One of the first two-and-a-half dimensional (2.5D) models proposed by Kippenhahn and Schlüter (1957) (KS) presented an idealized prominence as an infinitely thin and long vertical sheet of dense material standing on its edge above a solar PIL, supported in an arcade of dipped magnetic field lines oriented perpendicular to the PIL (Fig. 2a). Figure 2b, c represent a version of this model discussed by KS that is of particular interest because it is both analytic and stable. The arcade in this solution is quadrupolar, which enables dipped fields within an initially potential (current-free) magnetic field and so a stable environment for prominence formation (Fig. 2b). As the prominence mass accrues, the magnitude of the magnetic dip increases and a localized current sheet forms, producing an upwardly-directed Lorentz force that supports the prominence mass against gravity (Fig. 2c). Note that prominence mass is fundamental to the presence of currents in this particular model, in that the upward Lorentz force associated with the current sheet is directly balanced by gravitational forces acting on the prominence mass $\rho$: i.e., $\rho *g = B_x(x=0)*[B_z]$, where $[B_z]$ is the jump across the current sheet as $B_z$ switches sign (Anzer 1969). Thus, in the limit of prominence mass approaching zero, the current sheet vanishes and the configuration reverts to its initial potential *dipped arcade* configuration.

**Fig. 3:**
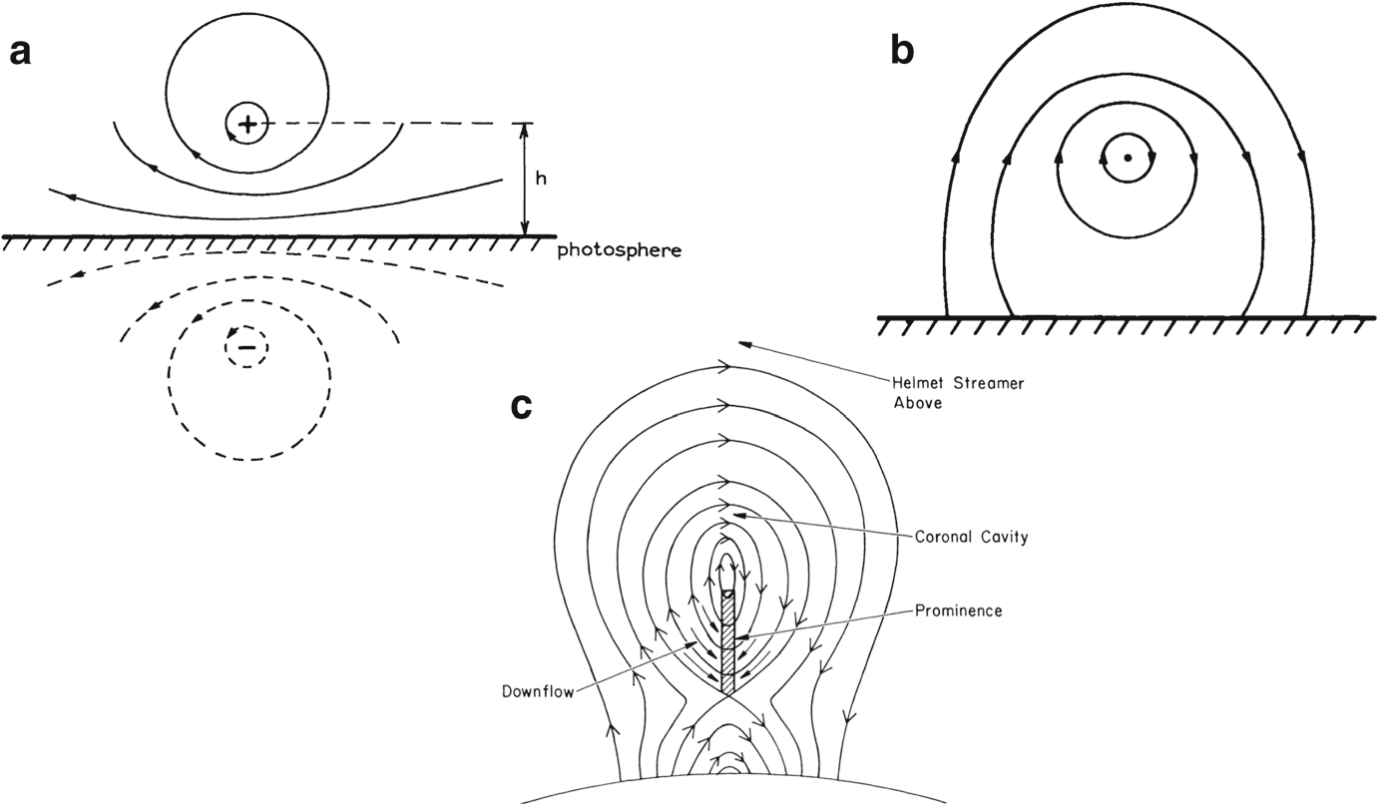
Cross sections of 2.5D flux-rope type prominence models. **a** Kuperus and Raadu model field due to a current filament a distance *h* above the photosphere (with image currents), and **b** the same embedded within a background potential field. Image reproduced with permission from Kuperus and Raadu (1974), copyright by ESO. **c** Summary configuration with the addition of X-line below prominence. Note that the current associated with the prominence axis is pointing *into* the page. Image reproduced with permission from Pneuman (1983), copyright by D. Reidel

Another 2.5D model by Kuperus and Raadu (1974) (KR) considered the prominence to be aligned with a “current filament”, detached from the photosphere and surrounded by a system of closed field lines (Fig. 3a). Induced currents at the photosphere repel those of the current filament, creating an upwardly-directed expansion force that provides support and stabilization against gravity for the prominence mass. A background field possessing horizontally-directed magnetic fields provides an additional confining downward force (e.g., Fig. 3b). In this scenario, the height at which the structure finds equilibrium can be affected by the mass of the prominence, and indeed, the authors argued that mass draining could lead to an increase in that height and potentially a loss of equilibrium (see also discussion in Low 1996). Since the prominence is magnetically-dominated, a component of the prominence magnetic field aligned parallel to the currents is needed to avoid destabilizing magnetic pinch effects. In the limit of prominence mass approaching zero, therefore, an equilibrium may exist in the form of a force-free magnetic *flux rope* containing field-aligned currents (Kuperus and Raadu 1974).

It is important to note that the prominence mass in the KR model may deepen the helical magnetic dips it accumulates in, creating a localized current sheet akin to that of the KS model with its own upwardly-directed Lorentz force. Thus, if we zoom in to the vicinity of the prominence itself, the models are actually quite similar: compare for example Fig. 2a to the central portion of Fig. 3c. The difference that does exist is one of reflection—in the KS model, the dipped magnetic field of the prominence runs left to right and the current is pointing out of the page, while the situation is the opposite for the KR model. As we discuss below, this difference must be considered in the context of the larger-scale magnetic equilibrium structures that encompass the prominence.

### Inverse versus normal magnetic fields

A key paradigm in the historical development of prominence models was that of *inverse* vs. *normal* magnetic configurations (Leroy 1989). This nomenclature represents a concept where the direction of horizontal magnetic fields associated with the prominence dips is compared to the magnetic polarities at the photosphere below. If the field in the prominence dip is oriented so that it points from positive to negative photospheric polarity, as would be the case in a simple arcade, it is referred to as *normal*. If it points from negative to positive polarity, it is called *inverse*. Early observations noted that an inverse configuration was in fact prevalent in prominences (Leroy et al. 1984), with 75–90% of prominences observed to have magnetic field oriented in a direction opposite to that expected from a potential field extrapolation (Démoulin 1998). Those oriented in the same direction, i.e., possessing a normal configuration, tended to be associated with stronger field active regions (Bommier et al. 1994).

**Fig. 4:**
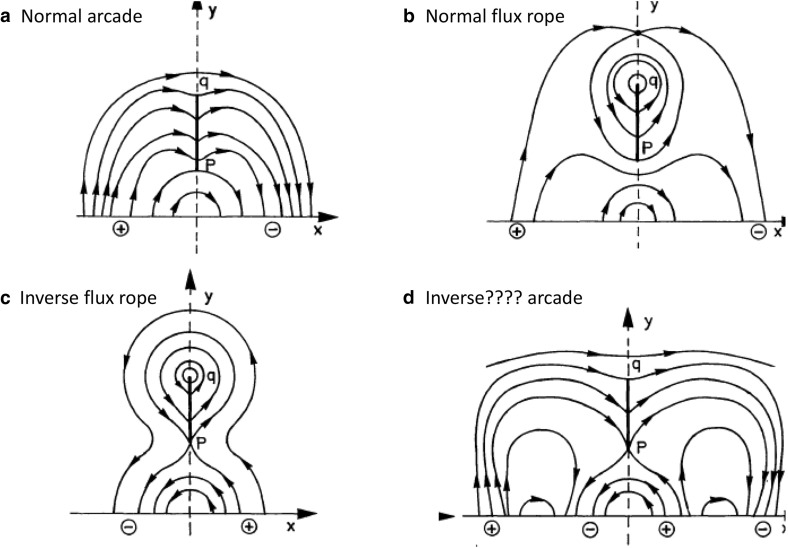
Examples of inverse and normal magnetic configurations, for both dipped arcade and flux rope models. “????” in panel **d** indicates the ambiguity discussed in the text. Adapted from Malherbe and Priest (1983)

Considering for now the bipolar versions of the KS and KR models shown in Figs. 2a and 3c, we see that the former represents a normal configuration and the latter an inverse one. However, it is not generally true that dipped arcades always have normal configuration and flux ropes always have inverse configurations. As was demonstrated by Malherbe and Priest (1983) and several other authors (Amari and Aly 1989; Démoulin and Forbes 1992; Démoulin and Priest 1993; Low and Zhang 2004), inverse arcades and normal flux ropes are also possible, even in 2.5D (see Fig. 4). Moreover, equating the KS model with the normal configuration and the KR model with the inverse configuration (as has often been done in the literature) can lead to confusion. Note that the quadrupolar model shown in Fig. 2c—from the original Kippenhahn and Schlüter (1957) paper—is identical to Fig. 4d—which was presented by Malherbe and Priest (1983) as an example of an inverse “KR” field.

But is that quadrupolar arcade model in fact an inverse field? An extrapolation of its photospheric field should arguably identify it as a normal prominence, since the direction of the dipped magnetic fields is the same as for the equivalent potential field (Fig. 2b). If instead the prominence fields are compared to a potential field extrapolation using only the bipolar field directly beneath the prominence, it would be characterized as inverse (Démoulin and Priest 1993). Although this latter approach is effectively that used in observational surveys of prominences (e.g., Leroy et al. 1984), realistic magnetic boundary conditions for prominence models may include curved or even multiple PILs. Thus, while inverse vs. normal is a fairly straightforward concept for 2.5D bipolar magnetic configurations, it becomes ambiguous for more complex fields.

The dichotomy that is perhaps more meaningful, and that we now explore further in 3D, is that of arcade vs. flux rope. A dipped (and in 3D, sheared) arcade is topologically equivalent to a potential arcade. This clearly distinguishes it—at least *theoretically*—from a flux rope model, which possesses magnetic field lines that wrap around each other in a manner not obtainable to the potential field. As we will see, the challenge is to clearly distinguish these two topologies from each other *observationally*.

### Sheared-arcade versus flux-rope models

#### Sheared-arcade models

The magnetic structure shown in Fig. 2c is a special case of a stable, dipped magnetic arcade with a current sheet that can be directly related to a dipped, fully potential field (Fig. 2b). It has the advantage of presenting a scenario in which the prominence may form in pre-existing dipped magnetic field that is the natural consequence of a particular photospheric magnetic flux distribution (Démoulin and Priest 1993). However, this distribution is quadrupolar, while the majority of prominences are associated with bipolar regions.

2.5D, bipolar, dipped-arcade models for prominences are possible, including configurations that are predominantly potential (Démoulin and Forbes 1992) or linear-force-free (Amari and Aly 1990; Démoulin et al. 1992) everywhere except for the prominence current sheet. However, such models beg the question of how the initial dipped configuration might arise. Since the critical element in a dipped bipolar arcade type topology is shear along the underlying neutral line, a logical approach would be to impose shearing motions on an arcade to create dipped fields. It is not possible, however, to form dips at the top of an arcade through shearing motions in 2.5D, because field lines expand and become flatter as magnetic shear increases (Klimchuk 1990; Amari et al. 1991).

**Fig. 5:**
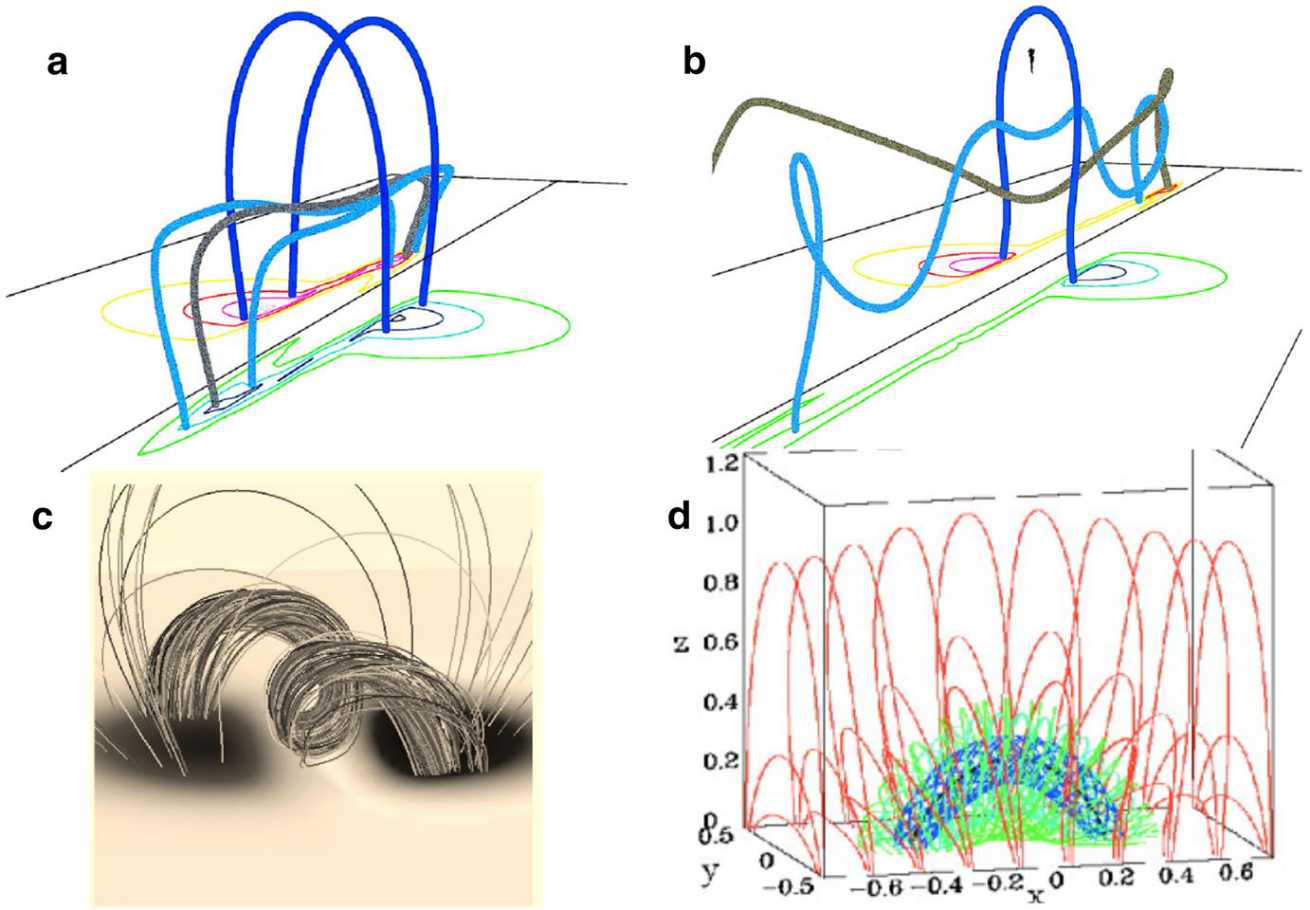
3D magnetic configurations with dipped field capable of supporting prominences. Field lines from DeVore and Antiochos (2000) **a** sheared arcade and **b** flux rope; **c**Amari et al. (1999) and **d** Fan and Gibson (2004) flux ropes. Images reproduced with permission, copyright by AAS

The case for a sheared arcade is much stronger in three dimensions, however, and (Antiochos et al. 1994) demonstrated how such a 3D force-free dipped arcade could be formed through shearing motions. In this case, the overlying arcade compresses the central part of the underlying sheared arcade locally, so that the dipped field is in the middle, rather than the top of the arcade (Fig. 5a). Moreover, the dipped fields in such a 3D sheared arcade are mostly inverse (Aulanier et al. 2002).

#### Flux-rope models

At this point we must pause and clarify our definition of a flux rope vs. sheared arcade. By a flux rope we mean anything in which magnetic fluxes wrap around a central axis (or axes, in the case of a spheromak). This leads to topologically-distinct magnetic sub-systems, such that e.g., quasi-separatrix layers (QSL—Priest and Démoulin 1995) form at steep gradients in field-line linkage, or bald-patch-separatrix surfaces (BPSS—Titov and Démoulin 1999; see also Fig. 9) manifest discontinuities between flux rope and neighboring arcade field lines. We will return to this topic in Sect. 3.2.

Mechanisms that have been proposed to create flux ropes include twisting motions at the photosphere (Priest et al. 1989), reconnection between sheared field lines (van Ballegooijen and Martens 1999), and emergence of pre-twisted fields from beneath the photosphere (Rust and Kumar 1994). Analytic flux-rope models evolved from 2.5 D, mostly force-free (Ridgway et al. 1991; Forbes and Isenberg 1991; Amari and Aly 1992; Low 1993; Schonfelder and Hood 1995) or magnetostatic configurations (Low and Hundhausen 1995), to 3D cylindrical (Titov and Démoulin 1999; Low and Berger 2003) and spheromak models (Lites and Low 1997; Gibson and Low 1998).

Further variety in 3D nonlinear force-free flux ropes has been enabled through numerical modeling. In particular, more realistic flux ropes embedded in overlying, near-potential arcades were formed through shearing followed by reconnection (DeVore and Antiochos 2000) (Fig. 5b), shearing followed by twisting motions or diffusive processes (Amari et al. 1999) (Fig. 5c), or emergence of twisted field into an overlying potential arcade (Fan and Gibson 2004) (Fig. 5d). Most of these models result in flux ropes that are not tightly wound, but rather possess between one and two turns about a central axis. Indeed, flux ropes that have more twist are prone to the kink instability, which may trigger eruption (Rust and Kumar 1996; Kliem et al. 2004; Török et al. 2004; Fan 2005). However, the kink instability may actually trigger a transition to a more tightly-wound topology, if such a topology represents a lower magnetic energy state. This has been demonstrated in a simulation where reconnections between an erupting, kink-unstable cylindrical flux rope and surrounding arcade fields resulted in a spheromak topology (Gibson and Fan 2008); arguably a quiescent spheromak field might arise in the case of a confined eruption (see, e.g., Török and Kliem 2005).

**Fig. 6:**
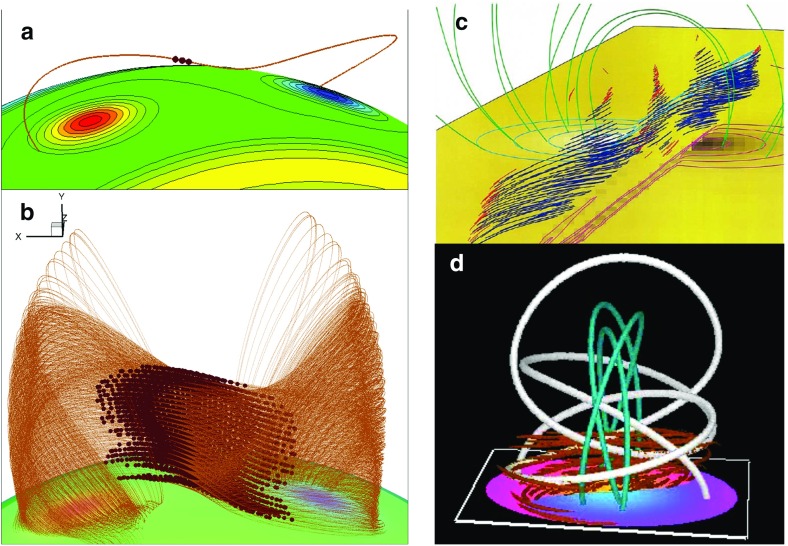
“Painting the dips” of a magnetic skeleton results in sheet-like prominences, independent of magnetic topology. **a** Demonstration of the small region this would represent for a sample, dipped field line within a flux rope field model, and **b** demonstration for many field lines within the rope (after Gibson and Fan 2006a). **c** Painted dips within 3D sheared-arcade model. Image reproduced with permission from Aulanier et al. (2002), copyright by AAS. **d** Painted dips within 3D analytic model of spheromak-type magnetic flux rope. Image reproduced with permission from Lites and Low (1997), copyright by Springer

#### Comparing to prominence properties

So, can the basic observed properties of prominences (as described in Sect. 2.1) distinguish between magnetic topologies? We have presented models that possess significant three-dimensional magnetic complexity, but that so far do not consider the thermodynamics and radiative transfer problems necessary to fully model the cool prominence in the hot corona (we will return to this in Sect. 4). However, since we expect the prominence mass to be localized in dipped magnetic field (we will revisit this assumption in Sect. 4.2.1), we can extract a proxy for the location of prominence mass by “painting the dips” within any modeled magnetic field. The justification for this approach is that localized cross-field currents supporting prominence mass do so without substantially deforming the larger-scale force-free magnetic skeleton (we will revisit this assumption in Sect. 4.2.2). Because the pressure scale height for the relatively cool prominences is much smaller than for coronal plasma, the prominence may only fill a very small portion at the lowest portion of each magnetic dip (and, depending on the local thermal environment, may not fill even that). The result will be bead-like condensations that collectively form the prominence (see discussion in Low 1982; also Fig. 6a, b).

Figure 6b, d show the loci of dipped magnetic field lines for models ranging from sheared arcade to cylindrical flux rope to spheromak flux rope (all of which have predominantly inverse configurations—see Sect. 2.3). From this we see that these disparate topologies all result in a sheet-like prominence structure even though there are no obvious sheet-like discontinuities in the surrounding magnetic skeleton. Counterintuitive though it may seem, even the highly twisted spheromak magnetic field results in a vertical sheet that makes only a slight angle to the underlying neutral line.

The models we have discussed so far have been idealized, with homogeneous, mainly bipolar photospheric boundaries. More realistic models would require solving a “mixed boundary problem” that takes into consideration both the prominence and the full complexity of the lower magnetic boundary. (See Démoulin et al. 1992 for a discussion of the historical development of the mixed boundary problem as applied to prominences.) Extrapolations from photospheric magnetic observations have explicitly incorporated a prominence in the form of an inserted flux rope in fitting linear (Aulanier et al. 1999) and nonlinear (van Ballegooijen 2004; Su and van Ballegooijen 2012) force-free models. In some cases, observations of coronal structures have also been used to constrain such magnetic extrapolations (Savcheva and van Ballegooijen 2009; Malanushenko et al. 2014). With this in mind, we now turn to other signatures of the magnetic structures that encompass prominences.

## Beyond bones: the prominence in context

In Sect. 2, we discussed how prominences (which we will also refer to as filaments when viewed against the solar disk) are observed to form above photospheric polarity inversion lines. Additional observed properties of their environment include the fact that they form within magnetically-defined filament channels, and that closed magnetic fields surround them, manifesting as an overlying helmet streamer, coronal cavity, and/or system of sheared, sometimes sigmoidal, coronal loops.

### Filament channels and chirality

**Fig. 7:**
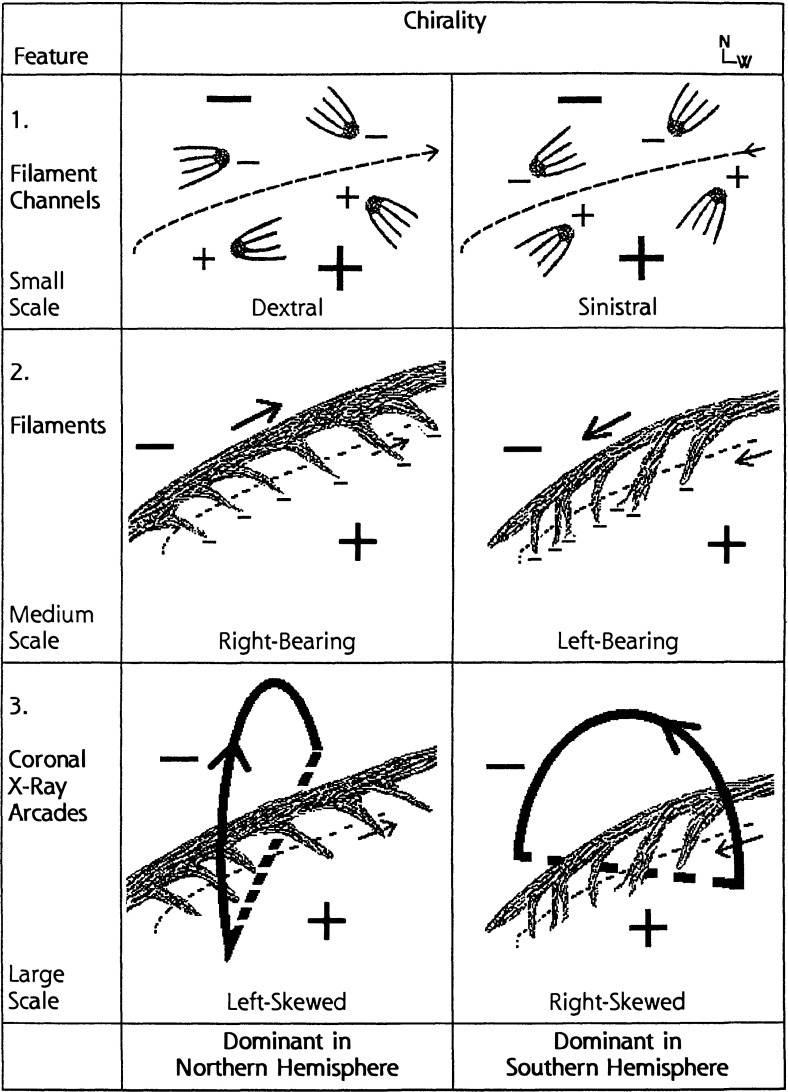
Chirality relations between prominence(filament) and surrounding environment. **1** Chromospheric fibrils observed in filament channels; **2** filament extensions (barbs) from central spine; and **3** overlying coronal loops. Note this is the classic sketch from Martin (1998b), copyright by ASP—for an updated sketch see Fig. 7 of Martin (2015). This recent review emphasizes that proper chirality determinations require adequate spatial resolution and/or flow patterns to aid in seeing the direction of filament threads, as well as consideration of the perspective from which a filament is viewed

Filament channels are regions where chromospheric fibrils are aligned on either side of a polarity inversion line (Fig. 7a). They are arguably “more fundamental than filaments” (Mackay 2015), since a filament channel may exist without a filament, but a filament cannot exist without a filament channel. The patterns formed by the fibrils as they relate to surrounding magnetic fields has been interpreted as a indication of magnetic chirality, or handedness (Martin 1998a). In particular, when viewed from the positive polarity side, if the fibrils on the negative side of the filament channel extend to the right, they are referred to as “dextral”, and if they extend leftward, they are referred to as “sinistral”.

**Fig. 8:**
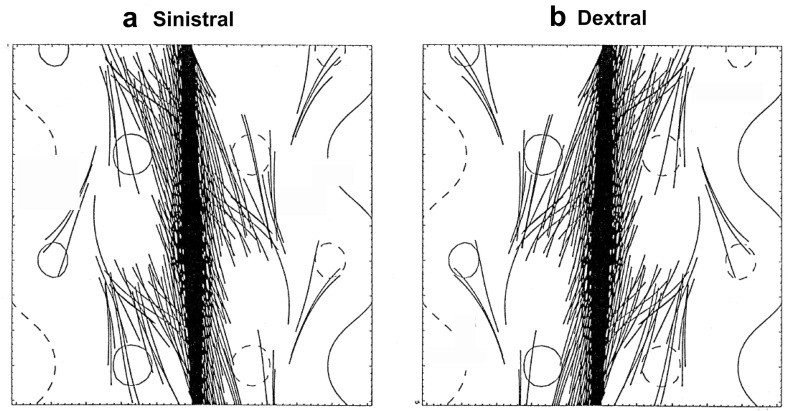
Adding parasitic polarities to the photospheric flux distribution results in a linear-force-free flux rope with filament barbs. Dark lines represent dips in magnetic field lines consistent with **a** left-handed (sinistral) and **b** right-handed (dextral) chirality barbs. Image reproduced with permission from Aulanier and Démoulin (1998), copyright by ESO; see also Aulanier et al. (1998)

**Fig. 9:**
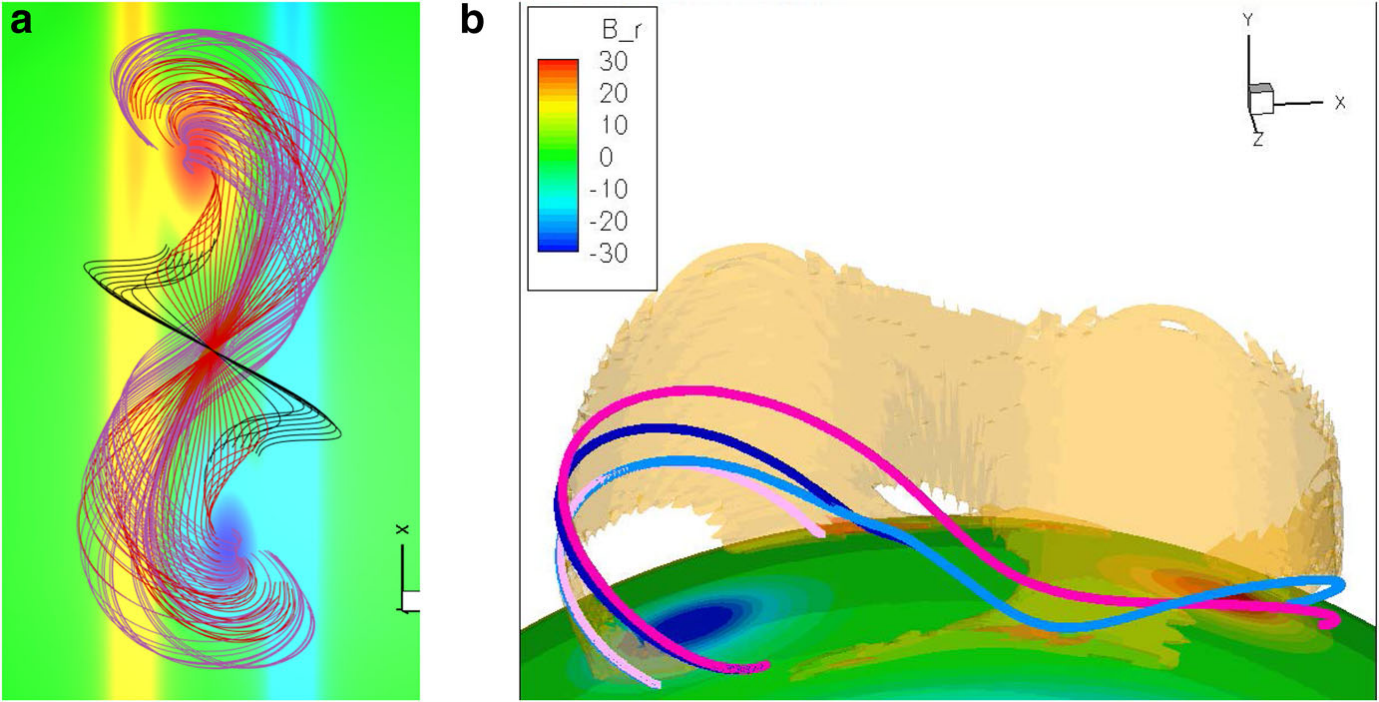
Equilibrium magnetic flux rope model and sigmoidal separatrix. **a** Demonstration of the oppositely directed field-line orientations relative to the PIL manifested at the bottom versus top of a right-handed helical flux rope. Magenta field lines represent the bald-patch-separatrix surface (BPSS) and trace out the bottom of the helix as it grazes the photosphere, while black field lines represent the oppositely oriented field lines at the top of the flux rope (after Fan 2017). **b** Current sheet (isosurface) that forms under dynamic (but not eruptive) perturbation, leading to interchange reconnections between BPSS (light-blue) and adjacent arcade (dark-blue) field lines and resulting in reconnected sigmoidal/arcade field lines (dark pink/light pink). Image reproduced with permission from Gibson and Fan (2006a), copyright by AGU

Filament *barbs* are also observed to extend from the central spine (Fig. 7b). The tendency of these to bear left or right (as viewed from the positive polarity side) is another measure of chirality that consistently matches that observed for the filament channel fibrils. These barbs are associated with small regions of opposite, or “parasitic” magnetic polarity (Martin 1998a). Aulanier and Démoulin (1998) built a linear force-free flux rope with a periodic boundary to demonstrate that the introduction of parasitic polarities to a larger-scale bipolar photospheric flux distribution resulted in magnetic dips organized in a manner reminiscent of such barbs extending out from the central sheet (Fig. 8). However, there is some controversy regarding the magnetic structure of barbs, and how these relate to flows in prominences (Chae et al. 2005). We will return to the question of flows vs. field in Sect. 4.1).

Finally, the coronal loops observed above filaments have their own indication of chirality. In this case, however, the overlying structure skews at an angle that opposes the underlying filament and fibril structures. In particular, as Fig. 7c illustrates, a right-handed (dextral) filament channel always develops beneath a left-skewed coronal loop system, and vice versa (Martin and McAllister 1996).

Taken together, the observed chirality properties of filament channel, filament, and overlying arcades are easily reconciled with a magnetic flux rope topology; in particular, the overlying skewed arcade is a natural consequence of a helical system where the tops of winding field lines have the opposite orientation vs. the bottoms relative to the underlying PIL (Fig. 9a). Similarly, Gibson and Low (2000) described how a spheromak topology could also result in these chirality properties. However, a large amount of twist is not necessary and indeed simulations of what many would call sheared arcades (possessing field lines that wind less than a full turn about a central axis) also result in oppositely directed skew for overlying field lines vs. lower-lying, dipped field lines (e.g., Welsch et al. 2005). For all of these models, the opposite orientations observed for filament vs. overlying arcade does not require opposite signs of magnetic helicity (see discussion in DeVore and Antiochos 2000).

### Sigmoids and separatrices

**Fig. 10:**
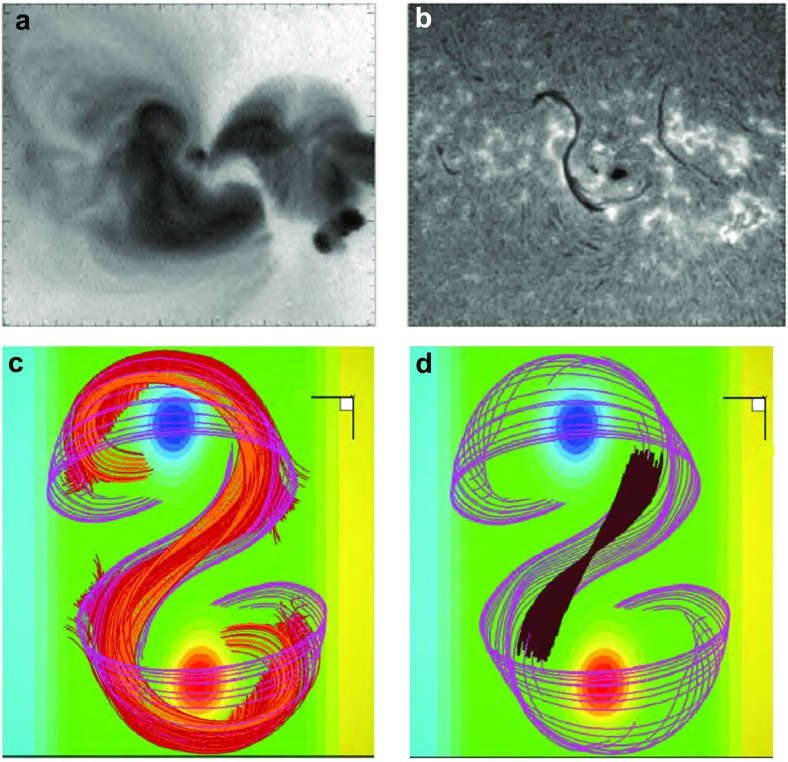
A flux-rope model filament lies above and within the BPSS identified with a SXR sigmoid. **a** Yohkoh SXT (inverse color scale) and **b** Big Bear Solar Observatory (BBSO) observations of a SXR sigmoid and H$\alpha$ sigmoidal filament. Image reproduced with permission from Gibson et al. (2002), copyright by AAS. **c** Flux rope with BPSS (purple) and current-sheet-intersecting field lines (red/orange). Orange field lines in **c** have dips, and **d** paints the centers of these and other dipped field lines up to a prominence scale height (brown). Image reproduced with permission from Gibson and Fan (2006a), copyright by AGU

Soft-Xray (SXR) “sigmoids” are active regions with a characteristic S or inverse-S shape (Rust and Kumar 1996) (Fig. 10a). These regions are prone to eruption (Canfield et al. 1999; Glover et al. 2000), leading to the interpretation that the sigmoidal shape indicates non-potentiality, or energization. Sigmoids range from regions of sheared loops which collectively create an S- or inverse-S-shaped pattern, to sharply defined individual sigmoidal loops. The former may be long-lived, manifesting for multiple days, but the latter tend to be transient. Such transient sigmoid loops may exist stably for several hours before an eruption onset (Green and Kliem 2009; James et al. 2017). They also may appear and disappear without eruption over the course of several days for a given region (Gibson et al. 2002).

Filaments are often associated with SXR sigmoids, and may exhibit a sigmoidal shape as well (Aurass et al. 1999; Gibson and Low 2000; Pevtsov 2002; Gibson et al. 2002) (Fig. 10b). Unlike the overlying arcades observed by Martin and McAllister (1996), SXR sigmoids show the same direction of skew vs. the PIL as associated filaments, such that S (inverse-S) shapes are associated with positive (negative) chirality (Pevtsov et al. 1997; Green et al. 2007). An examination of Fig. 9a) in comparison to Fig. 8 reveals that this implies, at least for the flux-rope model, that the SXR sigmoid must be formed from field lines tracing out the bottom of the helix, rather than the top.

Various models have been put forward to explain sigmoids that are consistent with this observed chirality relation, and we now briefly highlight some representative examples (see also Gibson et al. 2006a; Green et al. 2007 for further discussion of these and other models). A combination of magnetic flux emergence and diffusive evolution may explain the long-lived sigmoid in terms of a sheared arcade of “J”-type loops, which then may reconnect to form an erupting flux rope and associated transient sigmoid (van Ballegooijen and Mackay 2007; see also Amari et al. 2000; Moore et al. 2001; Kusano 2005). Alternatively, an eruption in which a pre-existing rope kinks downward could create sigmoidal current sheet enhancements of the right orientation (Kliem et al. 2004).

Not every sigmoidal loop erupts, however, and other models address how they may form in quiescent magnetic structures in association with separatrix layers. In particular, QSLs (the areas where magnetic connectivity changes abruptly, see Sect. 2.4.2) are expected to be locations where current sheets form and reconnections occur (Démoulin et al. 1996; Titov et al. 2002; Aulanier et al. 2005). The nature and location of QSLs for flux ropes depends upon the rope’s topology. If the rope has an arcade beneath it (see Fig. 3d), the QSL will incorporate the line of magnetic X points that separate the rope from arcade, and be prone to current-sheet formation beneath the rope (Titov et al. 2003; Galsgaard et al. 2003). Simulations have demonstrated that magnetic field lines intersecting this sheet will form a sigmoid in the correct direction (Kliem et al. 2004; Török et al. 2004). Indeed, the QSL may evolve from 2 J-shapes to an S-shape, as tether cutting reconnections continue to add twist to the rope and drive its slow rise (Fan 2012) (see also Aulanier et al. 2010; Savcheva et al. 2012b, c).

**Fig. 11:**
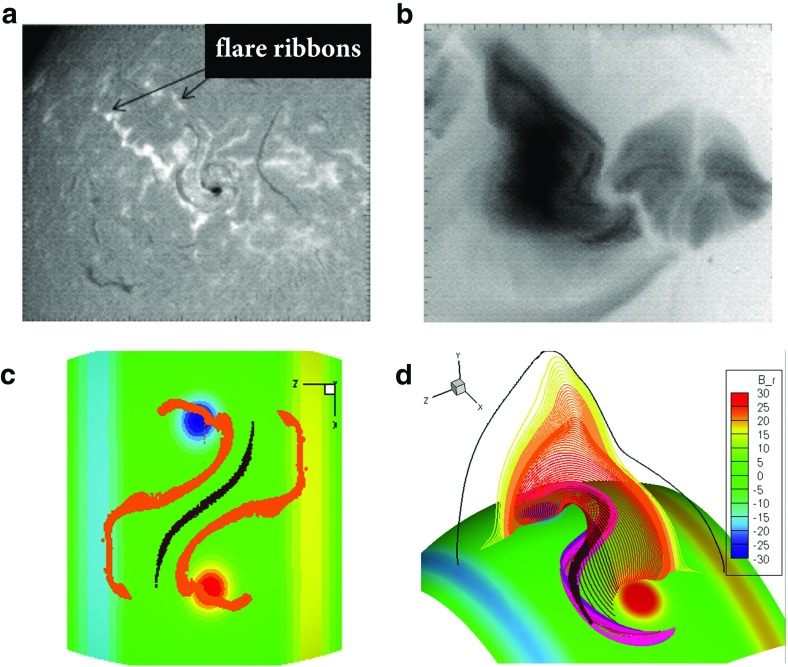
Partially-erupting sigmoids/filaments. **a** BBSO H$\alpha$ observations of flare ribbons surrounding a filament unaffected by the eruption and **b** Yohkoh SXT (inverse color scale) observations of a sigmoid with overlying cusped field lines. **c** Footpoints of reconnected field lines during partial flux rope eruption, and **d** field lines surviving the eruption, including cusped field lines (yellow/orange) overlying the remains of a BPSS (purple) and dipped field (brown). Note that the Yohkoh/BBSO observations of the eruption shown here are of the same region, but two days prior to the non-erupting sigmoid shown in Fig. 10. Images reproduced with permission from [top] Gibson et al. (2002), copyright by AAS, and [bottom] from Tripathi et al. (2009), copyright by ESO

For more low-lying flux ropes, the QSL takes the form of a sigmoidal BPSS (see Sect. 2.4.2) consisting of field lines whose dips just graze the photosphere—forming the so-called “bald patch” (Titov and Démoulin 1999; Low and Berger 2003). As Fig. 9b) illustrates, current sheets form at the BPSS leading to interchange reconnections between J-type arcade and S-type flux-rope field lines (Gibson et al. 2004; Gibson and Fan 2006a). The existence of BPSS-associated sigmoidal loops has been demonstrated observationally by Green and Kliem (2009), who analyzed the transition from J-shaped loops to a sharply defined sigmoidal loop, showing that it had 3 PIL crossings (as expected for a BPSS) and remained visible for several hours before erupting.

Moreover, in some cases a sigmoid will survive eruption, along with part of a filament (Pevtsov 2002; Green and Kliem 2009; Tripathi et al. 2009). This can be explained via the bifurcation of an erupting flux rope (Gilbert et al. 2000; Gibson and Fan 2006b) (Fig. 11). In this case, some portion of the sigmoid associated with the BPSS naturally survives the eruption because it lies below the filament (see, e.g., Fig. 10c, d), and Green and Kliem 2009 for further discussion.)

**Fig. 12:**
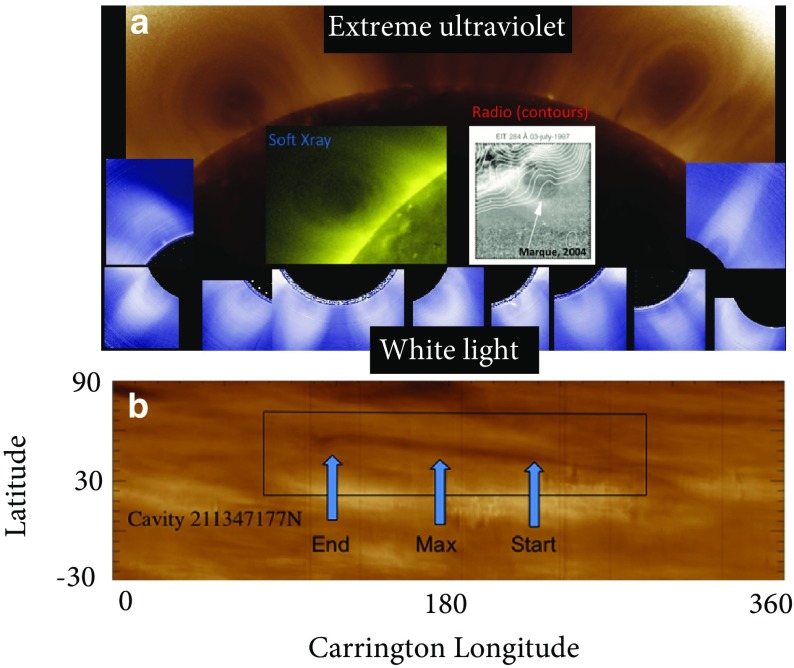
Cavities are ubiquitous and three-dimensional. **a** Collage of cavities observed over a broad range of wavelengths. Extreme ultraviolet (EUV) observations from Solar Dynamics Observatory/Atmospheric Imaging Assembly (SDO/AIA); SXR from Hinode X-ray Telescope (XRT); white light images from Mauna Loa Solar Observatory Mk4 K-coronameter (MLSO/Mk4); Radio contours (Nancay) overlaid on Solar and Heliospheric Observatory EUV Imaging Telescope (SOHO/EIT) observations (Marqué 2004). Image reproduced with permission from Gibson (2014), copyright by IAU. **b** Carrington latitude versus longitude plot constructed from AIA coronal limb observations (cropped to emphasize northern latitudes), showing the presence of an tunnel-like cavity extended in longitude. Image reproduced with permission from Karna et al. (2015b), copyright by AAS

### Cavities and flux surfaces

An examination of Fig. 6 demonstrates how—regardless of model—the prominence material is likely to fill only a fraction of the volume represented by its encompassing magnetic skeleton. When viewed along the prominence axis, this larger magnetic volume may become clearly detectable as a dark, elliptical cavity surrounding the prominence that extends along the line of sight (Engvold 1989; Tandberg-Hanssen 1995) (Fig. 12a). The cavity provides the silhouette of the “invisible man” (Gibson 2014) from which we can infer the full extent of the prominence magnetic skeleton. Cavity observations and models are reviewed comprehensively in Gibson (2015); we now briefly summarize the material presented there and in more recent publications.

**Fig. 13:**
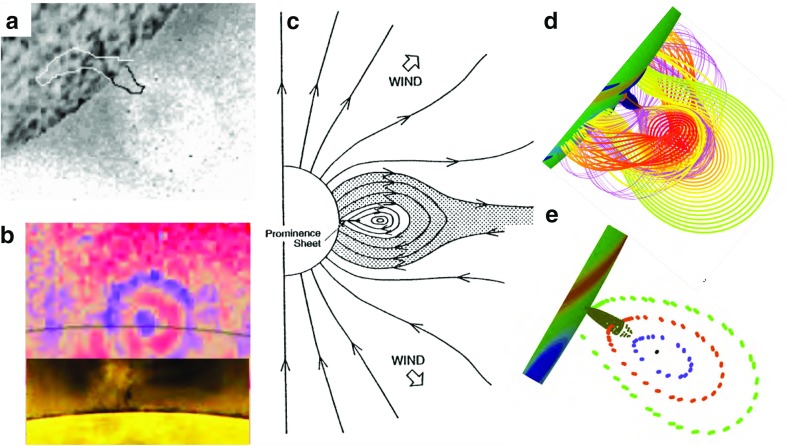
Hot cores and bullseye flows exist within larger cavity volume, perched atop prominences. **a** Yohkoh observation of long-lived hot SXR core (inverse image); location of H$\alpha$ prominence indicated by contour. Image reproduced with permission from Hudson et al. (1999), copyright by AAS. **b** (Top half) Bullseye line-of-sight flows observed by CoMP telescope within cavity; (bottom half) AIA 193 observations indicating presence of larger-scale cavity surrounding bullseye and central prominence (seen in absorption) beneath it. Image adapted from Bąk-Stęślicka et al. (2016). **c** Cartoon of cross section of flux rope embedded in closed and open fields. Image reproduced with permission from Low (1996), copyright by Springer. **d** Field lines within flux rope color-coded by temperature demonstrating the hot core lying above the dipped magnetic fields (brown) associated with prominence (from Gibson 2015); and **e** intersection of flux rope flux surfaces with plane of sky (colored dots). Image reproduced with permission from Forland et al. (2013), copyright by the authors

**Fig. 14:**
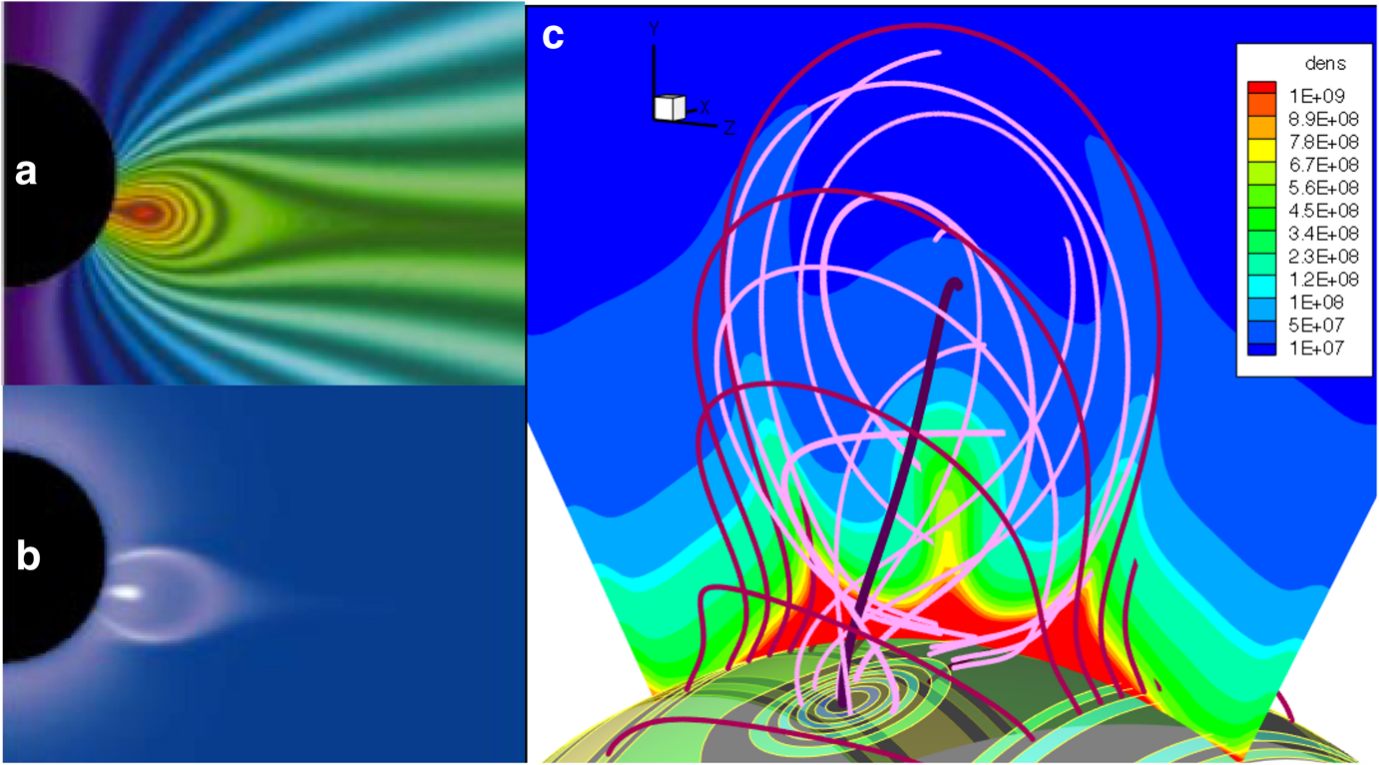
Simulations in which cavities arise from pre-eruption magnetic flux ropes. **a** Magnetic field lines and **b** line-of-sight integrated polarized brightness simulation. Image reproduced with permission from Linker et al. (2003), copyright by AIP. **c** Field lines with central cut showing cavity in density (after Gibson and Fan 2006a)

Cavities are particularly evident for large, quiescent prominences—especially those that make up the polar crowns—and may last for days, weeks, or even months (Gibson et al. 2006b; Karna et al. 2017). Observations of such regions over multiple days and from different viewing angles have demonstrated that they are tunnel-like, sometimes with a croissant-shaped morphology (Gibson et al. 2010; Karna et al. 2015a, b) (Fig. 12b). They have been detected at multiple wavelengths, from radio to white light to EUV and SXR (Saito and Hyder 1968; Waldmeier 1970; Saito and Tandberg-Hanssen 1973; Vaiana et al. 1973; Tandberg-Hanssen 1974; McIntosh et al. 1976; Straka et al. 1975; Kundu et al. 1978; Serio et al. 1978; Schmahl 1979) (Fig. 12a), indicating that they are regions of plasma depletion. However, their densities are still significantly higher than, for example, coronal holes; maximum cavity depletion is approximately 30–50% relative to surrounding streamer densities (Marqué 2004; Gibson et al. 2006b; Fuller et al. 2008; Schmit and Gibson 2011). Cavities have coronal temperatures, but may be multithermal along the line of sight (Kucera et al. 2012), and may have a hot, central core (Hudson et al. 1999; Habbal et al. 2010; Reeves et al. 2012). This core may lie above the prominence and in the center of the cavity, like a lollypop on a stick (Fig. 13a). Horn-like brightenings may lie at the bottom interface of this core (Régnier et al. 2011); these have been demonstrated to emanate from the prominence and connect it to the cavity (Schmit and Gibson 2013). Finally, swirling motions have been observed within the cavity (Wang and Stenborg 2010; Li et al. 2012; Panesar et al. 2013), and line-of-sight flows of coronal plasma follow the contours of the cavity boundary (Schmit et al. 2009), in some cases forming a bullseye pattern within the cavity and above the prominence (Fig. 13b; Bąk-Stęślicka et al. 2016).

The idea that cavities indicate the presence of pre-eruption flux ropes has been argued for many years (Pneuman 1983; Low and Hundhausen 1995; Low 1994, 1996, 2001) (Figs. 3c; 13c) and demonstrated with analytic and numerical models in which cavities may exist in equilibrium before an eruption (Gibson and Low 1998; Linker et al. 2003; Fan and Gibson 2006) (Fig. 14; see also Sect. 4.3). The magnetic flux surface at the boundary between rope and arcade naturally gives rise to the sharp, elliptical boundary, and swirling flows and bullseye patterns are similarly explained by the nested toroidal flux surfaces of the rope’s cross section (Fig. 13e). The relative locations of the prominence, hot central core, and surrounding large-scale cavity can all be explained in terms of the X-line/current sheet (Low and Hundhausen 1995), separatrix layers (Fan and Gibson 2006), and arched axial field lines within the rope (Schmit and Gibson 2014) (Fig. 13c, d). Explaining the density depletion in the cavity is somewhat more complicated; although force balance arguments can be invoked to require a strong axial magnetic field within the cavity to ensure total pressure continuity, this does not explain why the cavity is depleted in the first place (Low 1996). A variety of explanations are presented in Gibson (2015), including field-line length related thermodynamic effects and stability selection effects, and we refer the reader to that review for further discussion.

**Fig. 15:**
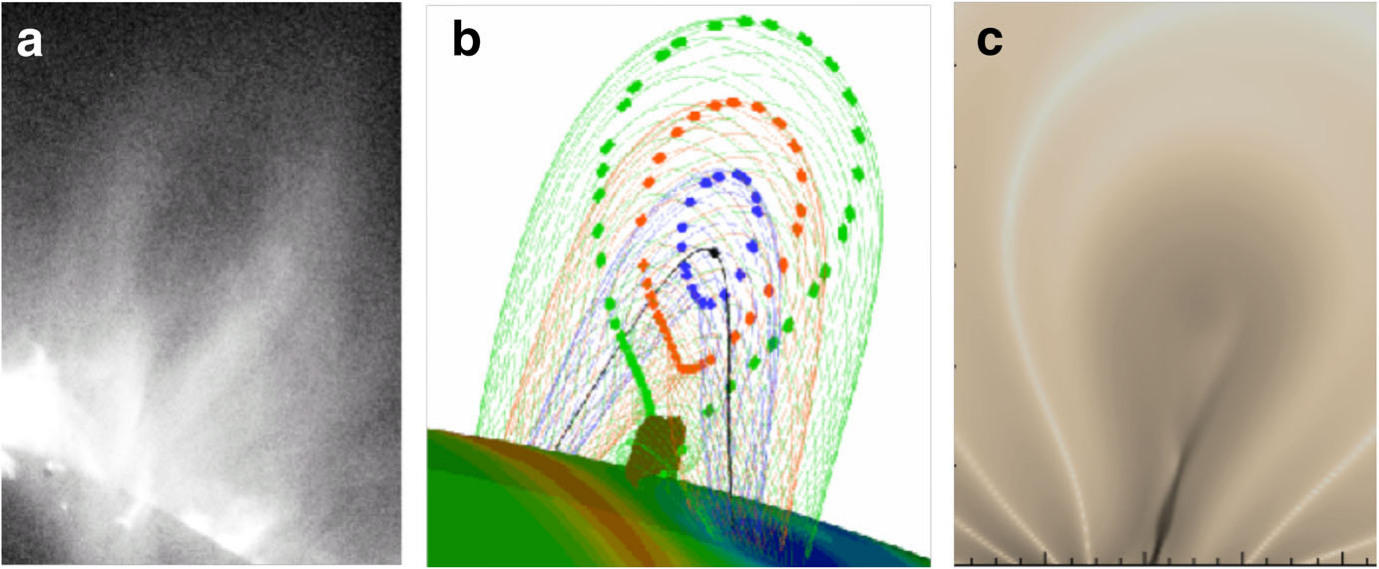
Cavities with teardrop morphologies are likely to erupt. **a** Hinode XRT observations of a cavity (01/11/2011 07:24UT) that erupted about 24 h later. Image courtesy K. Reeves. **b** Intersection of rope flux surfaces with plane of sky (colored dots). Image reproduced with permission from Forland et al. (2013), copyright by the authors—this may be compared to Fig. 13e which represents a flux rope with a BPSS, while the teardrop morphology of this figure represents a flux rope with an underlying X line. **c** Development of a teardrop morphology manifests as current enhancements (darker grey) within a torus-unstable erupting flux rope. Image reproduced with permission from Aulanier et al. (2010), copyright by AAS

Cavities can bodily erupt as CMEs (Yurchyshyn 2002; Vršnak et al. 2004; Gibson et al. 2006b; Maričić et al. 2009), and, like prominences, may be activated prior to eruption exhibiting a slow rise, narrowing, and enhanced substructure. Even before activation, cavity morphologies provide clues to their stability. Forland et al. (2013) found that the majority of AIA cavities in a survey based on 2010–2011 observations were prolate (taller than they were wide). Karna et al. (2015a) had similar results. Both studies found that a prolate, and especially a teardrop-shaped morphology in cavities was a strong predictor of eruption (Fig. 15a). A theoretical explanation for this might be that teardrop-shaped flux surfaces occur when an X-line lies beneath a flux rope (Fig. 15b, c), effectively acting as a “lit fuse” in which tether-cutting reconnections at the associated QSLs lead to flux-rope axis rise and, inevitably, at least partial eruption of both cavity and prominence (Aulanier et al. 2010; Savcheva et al. 2012a, b; Fan 2015).

Because of the elliptical flux surfaces intrinsic to a magnetic flux-rope topology, and the evolution of these surfaces towards a tear-drop shape as the rope loses stability, the flux rope model is well-suited to explaining the observed properties of quiescent cavities that eventually erupt. However, although there are many observations of cavities existing stably prior to eruption (Koutchmy et al. 2004; Gibson et al. 2006b; Régnier et al. 2011; Forland et al. 2013; Gibson 2015), there are also cases where a cavity (bubble) apparently forms during the eruption, especially in association with impulsive eruptions from active regions (Patsourakos et al. 2010a, b). In such cases, a flux rope may form during the eruption, and the pre-eruption prominence may be better modeled by a sheared arcade. The question is whether the (relatively) small spatial scales and short temporal scales of active-region vs. quiescent prominences imply different enough physical environments to generally result in different topologies (e.g., sheared-arcade vs. flux-rope), or whether these spatial/temporal factors affect our ability to observe signatures of flux ropes. The signatures may also differ: rather than dark cavities, active region flux ropes may manifest as bright, hot features (Fan 2012, 2017). Such “hot flux ropes” may be associated with prominences and are generally seen in conjunction with eruptions; some apparently exist in advance of the actual eruption (Zhang et al. 2012; Patsourakos et al. 2013), while others apparently form during it (see Nindos et al. 2015 and references therein).

A final comment before we leave the subject of prominence cavities. Infrared polarimetric observations from the Coronal Multichannel Polarimeter (CoMP) (Tomczyk et al. 2008) consistently demonstrate a lagomorphic (rabbit-head-shaped) linear polarization structure associated with cavities. This polarimetric signature has been demonstrated to be consistent with that expected by a magnetic flux rope topology (Bąk-Stęślicka et al. 2013). These observations are not sufficient on their own to rule out the sheared-arcade model, however. Distinguishing between these topologies using coronal polarimetry requires larger-aperture solar telescopes capable of observing closer to the limb and with light-gathering capacity sufficient to obtain magnetically-sensitive circular polarization measurements (Rachmeler et al. 2013). Existing linear-polarization observations do, however, rule out a spheromak topology for most cavities (although CoMP observations consistent with a spheromak topology have been obtained at least for one cavity Dove et al. 2011).

## Adding flesh and blood to the skeleton: incorporating dynamics and thermodynamics into prominence simulations

The discussion until now has implicitly assumed some qualities of our magnetic skeleton that we will now call into question: that it is unchanging (at least when eruption is not imminent) and that it must be consistent with a force-free equilibrium plasma. We are driven to question these assumptions by an ever-increasing complexity of observations which directly or indirectly challenge them, and also by a commensurate evolution in the sophistication of prominence simulations.

### Prominence dynamics

Prominences—even in their quiescent (non-eruptive) state—are intrinsically dynamic. The magnitude of these flows range from a few km/s to a few 10s of km/s (Parenti 2014). These flows are generally sub-sonic, and even more sub-Alfvenic—magnetosonic velocities in the lower corona are on the order of hundreds of km/s (Warmuth and Mann 2005). Such flows do not themselves challenge the assumption of force-freeness (which we will return to in Sect. 4.2.2). However, as we now discuss, some prominence motions appear to violate the assumption underlying our entire concept of a magnetic skeleton: that is, that the plasma is “frozen” into the magnetic field and so all flows must follow its local direction. This is an assumption which holds whether or not the plasma is force free, and is a property of highly-conductive plasmas.

**Fig. 16:**
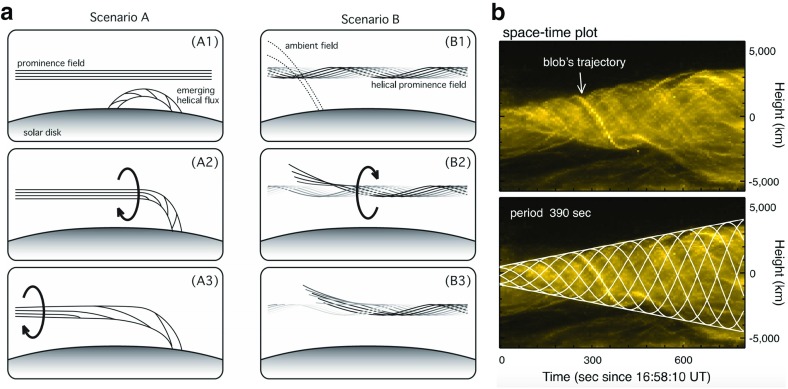
Dynamic evolution of the magnetic skeleton may manifest as prominence motions. **a** Schematic representations of how reconnections between the prominence magnetic flux system and emerging field (Scenario A) or neighboring, ambient field (Scenario B) might result in reconnection and the transfer of helicity and magnetic flux between the systems, with associated flows. **b** Observations of fine structures in a prominence spine in the Ca ii H line by Hinode/SOT are used to track the sinusoidal trajectory of a blob, which the authors interpret as evidence for Scenario B. Image reproduced with permission from Okamoto et al. (2016), copyright by AAS

Field-aligned flows do appear to be common. We have discussed swirling coronal plasma within cavities; cool prominence material is also observed to flow, streaming in both directions along the prominence spine (Schmieder et al. 1991; Zirker et al. 1998), or moving apparently helically about the apparently vertical prominence barbs in “solar tornados” (Pettit 1932; Orozco Suárez et al. 2012; Wedemeyer et al. 2013; Su et al. 2014). Caution must be exercised, however, since projection effects or oscillatory motions may lead to false indications of rotational flows in tornados (Panasenco et al. 2014; Martínez González et al. 2016; Levens et al. 2016; Schmieder et al. 2017). There is also some ambiguity about whether prominence flows are along stationary magnetic field lines, or whether they represent motions of the magnetic structure itself (thus, a dynamic skeleton). Evidence for the latter was presented by Williams et al. (2009), who observed a whole prominence rotating about its spine, and by Okamoto et al. (2016), who observed a small part of a prominence spine rotating and interpreted it as magnetic reconnection between flux systems (Fig. 16).

The deeper puzzle lies in the fact that although observations of prominence magnetic fields indicate they are near horizontal (Leroy 1988), vertical flows, both up and down, are observed in prominence barbs and within vertical striated “hedgerow” prominences (see Berger et al. 2008 and references therein). Observations of H$\alpha$ Doppler shift from the Meudon observatory indicated that the velocity vector is not aligned with the apparent vertical flows observed in intensity, but rather have a substantial horizontal component, consistent with flows tied to largely horizontal magnetic fields (Schmieder et al. 2010). This then begs the question of the cause of the apparent vertical structures and flows observed in intensity.

**Fig. 17:**
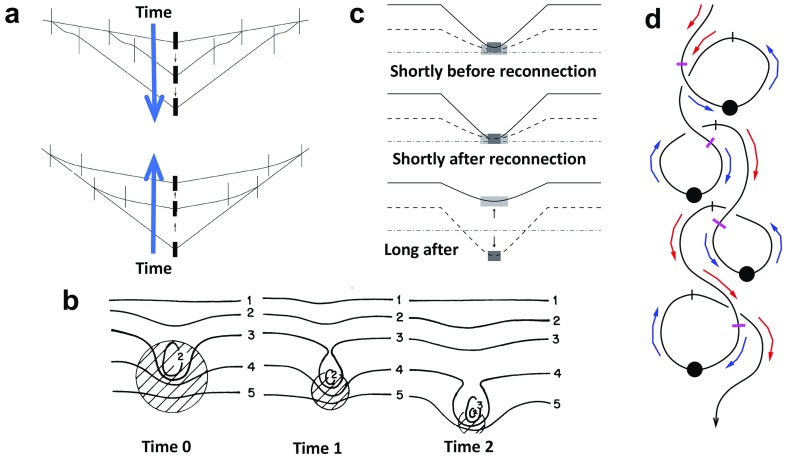
Vertical flows in prominence sheets are possible even when the local magnetic field orientation is horizontal. **a** Constant-speed descent (top) or ascent (bottom) may arise in KS-type prominence sheets that are in local equilibrium but out of equilibrium with their supporting magnetic structure. From Low and Petrie (2005). **b** Gravity acting on prominence plasma may lead to sagging field lines that reconnect to form a descending knot of closed field. From Lerche and Low (1980). **c** Reconnections at tangential discontinuities may pass a parcel of prominence mass downward across neighboring field lines whose dips collectively form a vertical structure. From Chae (2010); see also Petrie and Low (2005). **d** Tangled magnetic field lines may possess sufficient horizontal magnetic field to support prominence mass against free fall. From van Ballegooijen and Cranmer (2010). Images reproduced with permission, **a**, **c**, **d** copyright by AAS; **b** copyright by Springer

**Fig. 18:**
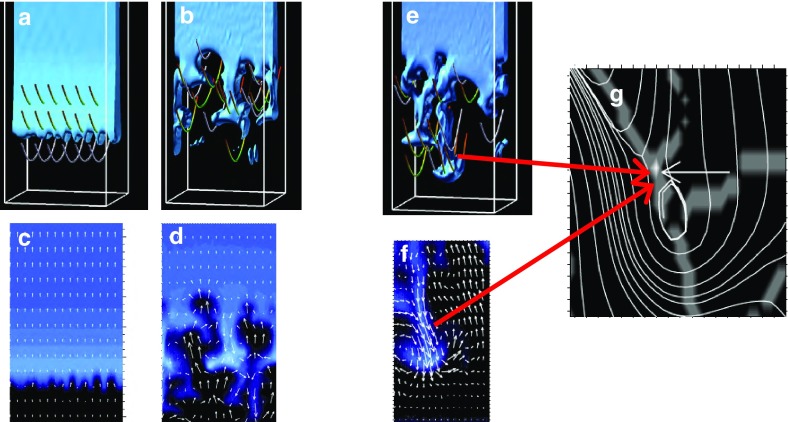
Vertical flows in prominence sheets associated with the Magnetic Rayleigh–Taylor (MRT) instability. **a** A KS-type prominence model is perturbed by **c** a low-density/high-temperature region at its base, resulting in **b**/**d** RT unstable upward flows (from Hillier et al. 2012a). **e**–**g** Reconnections in this evolving plasma result in downward acceleration of fast-moving, dense blobs (from Hillier et al. 2012b). Images reproduced with permission, copyright by AAS

Vertically-aligned structures have been modeled as the superposition of dips in horizontal field lines (Low 1982; Heinzel and Anzer 2001) (see also discussion in Parenti 2014 and references therein). Vertical flows in such structures—both upward or downward—may arise due to local response to an imbalance between magnetic tension and gravitational forces (Fig. 17a), or from reconnection within and/or between sagging, dipped field lines (Fig. 17b, c) (Lerche and Low 1980; Petrie and Low 2005; Chae 2010; Low et al. 2012). Alternatively, van Ballegooijen and Cranmer (2010) modeled the prominences as a vertical sheet of tangled magnetic fields in which prominence mass moves downward via a series of subsonic and supersonic flows separated by shocks and sonic points (Fig. 17d). All of these models are consistent with locally horizontal magnetic fields in the prominence, and downward velocities much less than expected for free fall, as observed.

The downward draining of prominence material has been argued to represent the first half of a “magnetothermal convection” cycle, where the second half involves the replenishment of prominence mass through upward plume-like flows from apparently hot, tenuous bubbles of plasma, observed to form beneath some quiescent prominences (Berger et al. 2011). These upflows have been argued to indicate a magnetic Rayleigh–Taylor (RT) instability between the coronal bubble and overlying prominence (Berger et al. 2010; Ryutova et al. 2010). The RT instability arises in general when heavy fluid lies above lighter fluid; such a situation can occur in a magnetized plasma, where, although total pressure (magnetic plus thermal) is required to be continuous across flux boundaries, thermal pressure is not. Magnetic fields actually play a stabilizing role, but only to perturbations parallel to the field (undular modes), and most strongly to short wavelengths. Perturbations perpendicular to the field result in the interchange of magnetic field lines. This has been demonstrated in a numerical simulation where a nonlinear perturbation to a KS dipped field model (see Sect. 2.2) resulted in upflows as field lines glide past each other (Hillier et al. 2011, 2012a) (Fig. 18a–d). Current sheets forming in the nonlinear phase of the RT instability then may lead to reconnections and downflows, completing the cycle (Fig. 18e–g).

Dudík et al. (2012) presented an alternative explanation for the plume upflows. These authors questioned the interpretation of bubbles and plumes as hot, arguing that they might rather be gaps in the prominence structure through which the background coronal emission became apparent. They demonstrated how a bubble consisting of arcade-type field lines might form below the dipped field of a linear-force-free flux rope perturbed by parasitic bipoles. They conjectured that the plumes were driven by reconnection at the magnetic separator outlining the bubble (see also Gunár et al. 2014). Further discussion of models of vertical flows in prominences, including a thorough review of prominence RT instability studies, can be found in Hillier (2018).

In summary—we cannot assume that our magnetic skeleton is static, or that flow lines necessarily indicate field lines. Prominence flows could also indicate a solid-body motion of the skeleton itself (e.g., Fig. 16), a breaking of it due to reconnection or deformation of it by gravity (e.g., Fig. 17), or an instability driving the rearrangement of magnetic field lines within it (e.g., Fig. 18). The skeleton itself might even be “leaky”, with the diffusion of neutral atoms playing a role in prominence mass loss and dynamical instabilities (Mercier and Heyvaerts 1977; Gilbert et al. 2002; Khomenko et al. 2014), and/or the frozen-in condition spontaneously and recurringly breaking down (Low et al. 2012; Low and Egan 2014). This complicates the interpretation of observations, and motivates the development of comprehensive 3D numerical MHD simulations that explicitly take into consideration the dynamics—and as we now discuss, the thermodynamics—of the prominence system.

### Prominence thermodynamics

At the risk of mixing metaphors: What makes a lake or a river? Topography clearly plays an essential role, but so does climate. Without frequent precipitation we are left with a dry river bed, or an empty lake. Similarly, a magnetic structure with a locus of dipped field lines may be conducive to supporting a prominence in equilibrium, but ultimately thermodynamics determines whether one actually forms.

But does this formation actually require an equilibrium? And, just as water may change the topography of the river bed it flows through, might the prominence plasma deform the topography of the magnetic field? It is clear that the 3D magnetic field that supports the prominence and the thermodynamic environment that enables its formation must be considered together if we are to answer these questions.

#### Beyond equilibrium

One dynamic approach developed to model prominence formation explicitly depends upon thermal nonequilibrium (TNE) (Antiochos and Klimchuk 1991; Antiochos et al. 1999, 2000). Here, heating at field-line footponts results in catastrophic cooling and condensation and forms a prominence that never reaches a static equilibrium, but rather is the product of an ongoing cycle of formation, motion, and destruction. Such a simulation raises the question: do we even need dipped magnetic fields for the prominence to form? To quote the pithy Karpen et al. (2001) abstract, “No.” Long, flat field lines are sufficient for a steady-state solution. For further discussion of this and other prominence formation models, we refer the reader to the review by Karpen (2015), and references therein.

**Fig. 19:**
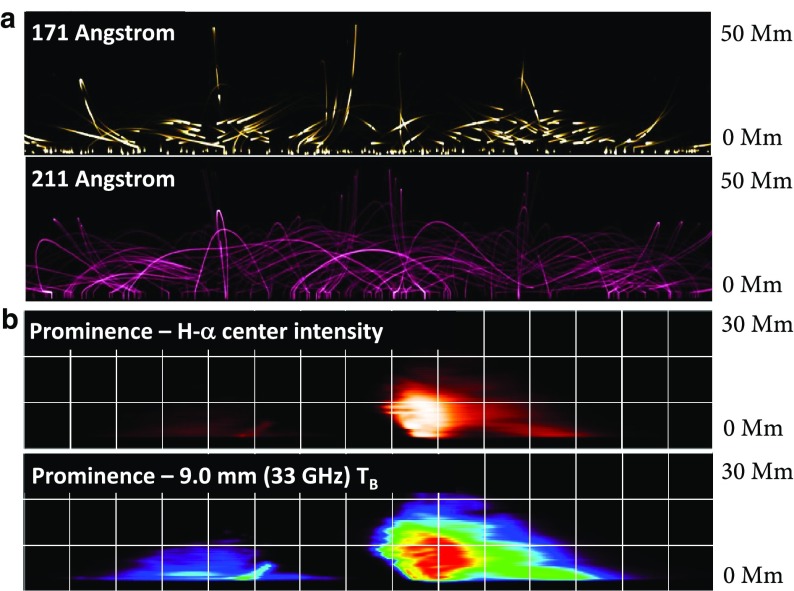
Fine structure associated with prominences can be modeled within a 3D magnetic structure. **a** Snapshots of time-varying coronal emission at (top) 171 Å and (bottom) 211 Å, for a 1D multithread simulation of TNE in a 3D sheared arcade (from Luna et al. 2012). **b** Prominence fine structure in (top) H$\alpha$ emission and (bottom) 9.0 mm (33 GHz) brightness temperature determined from plasma in 1D hydrostatic equilibrium along field lines within a 3D NLFF magnetic model (from Gunár et al. 2016). Images reproduced with permission, copyright by AAS

In modeling the full prominence magnetic skeleton, thermodynamic effects may be introduced by applying 1D simulations of plasma evolution to sampled magnetic field lines within a 3D magnetic model. In this way Lionello et al. (2002) demonstrated the condensation of cool material in magnetic dips of a 3D magnetic flux rope, solving steady-state hydrodynamic equations along field lines with conduction, heating, and radiative losses included. Luna et al. (2012) used TNE to investigate the fine-structure dynamics of the prominence and associated coronal emission, simultaneously solving TNE upon multiple field lines within a sheared arcade (Fig. 19a). Similarly, Schmit et al. (2013) applied TNE to sample field lines in a 3D flux rope model, providing clues to the prominence-cavity connection.

Although it did not explicitly model prominence formation, the Whole-Prominence Fine Structure (WPFS) model (Gunár and Mackay 2015a) also established a plasma distribution within a 3D non-linear force free (NLFF) magnetic field model (Mackay and van Ballegooijen 2009). This was conceptually similar to “painting the dips” as in Fig. 6, but done in a physically self-consistent way by imposing a semi-empirical temperature structure including a prominence-corona transition region and solving for hydrostatic balance (Gunár et al. 2013). This then allowed the generation of synthetic observations of, e.g., hydrogen emission through 1D radiative transfer calculations along multiple lines of sight (Heinzel et al. 2015), as well as brightness temperature and optical thickness at submillimeter wavelengths (Fig. 19b; Gunár et al. 2016). This is an intrinsically static approach; however, the time evolution of fine structures was also obtained in the form of a sequence of quasistatic equilibria by allowing the photospheric magnetic boundary to change, and with it the 3D NLFF magnetic skeleton (Gunár and Mackay 2015b). Thus, the WPFS approach obtained dynamics by evolving the magnetic skeleton itself, while the TNE approach was based on an unchanging magnetic skeleton, with flows due entirely to thermodynamics.

#### Beyond force-free

In Sect. 4.1, we discussed prominence dynamics that appear inconsistent with either thermally-driven flows along field lines or boundary-driven evolution of the skeleton itself, and discussed the possibility that some of these were due directly or indirectly to gravitational forces. This would violate the force-free assumption commonly made for coronal physics—that is, that magnetic forces self-balance and that thermal/gravitational forces are tiny in comparison.

As discussed in Sect. 2.1, observations of prominence plasma and magnetic fields have motivated this assumption for decades: early calculations found plasma $\beta$, the ratio of thermal to magnetic pressures, of order $\approx 0.02$, based on average magnetic field measurements of $\approx 10$ Gauss and pressure of $\approx 0.1\mathrm{\ dyne\ cm}^{-2}$ (Rust 1967). This study found field strengths of $\approx 5$–10 Gauss for most quiescent prominences, and higher strengths (60 Gauss) in active-region filaments, although the median values for active-region filaments were also in the 5–10 Gauss range. More recent observations have found patches of substantially higher magnetic field strengths (60–80 Gauss) in quiescent prominences (Casini et al. 2003), and even higher magnetic field strengths, on the order hundreds of Gauss, in active-region filaments (Kuckein et al. 2009). Luna et al. (2018) surveyed nearly 200 filaments near solar maximum, including both active region and quiescent filaments, and used prominence seisomology to establish *minimum* magnetic field strengths ranging from 2–38 Gauss, with an average of 17 Gauss. This study also found that the minimum magnetic field strengths did not differ significantly for quiescent vs. active-region filaments. All of these observations support the concept of *generally* force-free prominences.

It is clear, however, that magnetic field strengths are not homogeneous across the prominence, and similarly that their masses may vary significantly. Assuming magnetic field strengths of a few Gauss and modeling a massive, well-developed prominence, Anzer and Heinzel (2007) found plasma $\beta$ could be of order unity or higher in quiescent prominences. Such considerations, combined with the aforementioned prominence dynamics observations, motivate us to to consider the implications and interpretation of non-force-freeness in prominence simulations.

Hillier and van Ballegooijen (2013) examined the effects of mass loading an initially force-free, 2.5D flux rope, and relaxing it to a new MHD equilibrium. They found that increasing mass and/or plasma $\beta$ led to deformation of the magnetic field lines, in particular pulling the flux rope axis down. They argued that the most prominence-like distribution of mass (sheet-like and high density) occurred for cases where gravity was balanced by the additional magnetic tension force introduced by the deformation, and where $\beta$ (and with it, the thermal pressure gradient) possessed moderate values ($\approx 0.1$). They thus concluded that magnetic forces were fundamental to the prominence support; however, thermal and gravitational forces could not be considered a small perturbation to the system.

Terradas et al. (2015) undertook a similar study using a 3D sheared arcade and found that the global morphology of the modeled prominence depended on plasma $\beta$, with lower $\beta$ plasma supporting detached prominences and higher $\beta$ resulting in mass extending down to the photosphere, in a manner reminiscent of hedgerow prominences. For low $\beta$ cases, they found evidence of Magnetic Rayleigh-Taylor (MRT) dynamic behavior. Terradas et al. (2016) then studied the dynamic evolution of mass inserted into a 3D force-free flux rope (Titov and Démoulin 1999). Here again the magnetic structure was pushed downward by the mass and the field lines slightly deformed, with the dips associated with flux-rope twist providing support against gravity in a low-$\beta$ regime. Interestingly, the MRT instability was suppressed and the prominence was structured horizontally along the rope axis, rather than vertically, due to the Kelvin–Helmholtz shear instability. The authors concluded from these two studies that horizontally-structure prominences may be more consistent with a flux-rope topology, while vertically-structured prominences may be more consistent with a sheared arcade.

In summary—we cannot assume that the prominence is in equilibrium, or that it is fully force-free. Prominence formation and evolution respond to an ever-changing thermodynamic and magnetic environment, and the environment in turn may be affected by the prominence plasma.

### The Full Monty: prominence formation in 3D MHD

Finally, we turn to two recent MHD simulations that provide self-consistent representations of the prominence forming within a 3D magnetic skeleton. Both are flux rope models, and both explicitly treat the energy equation including conduction, radiative cooling, and coronal heating.

**Fig. 20:**
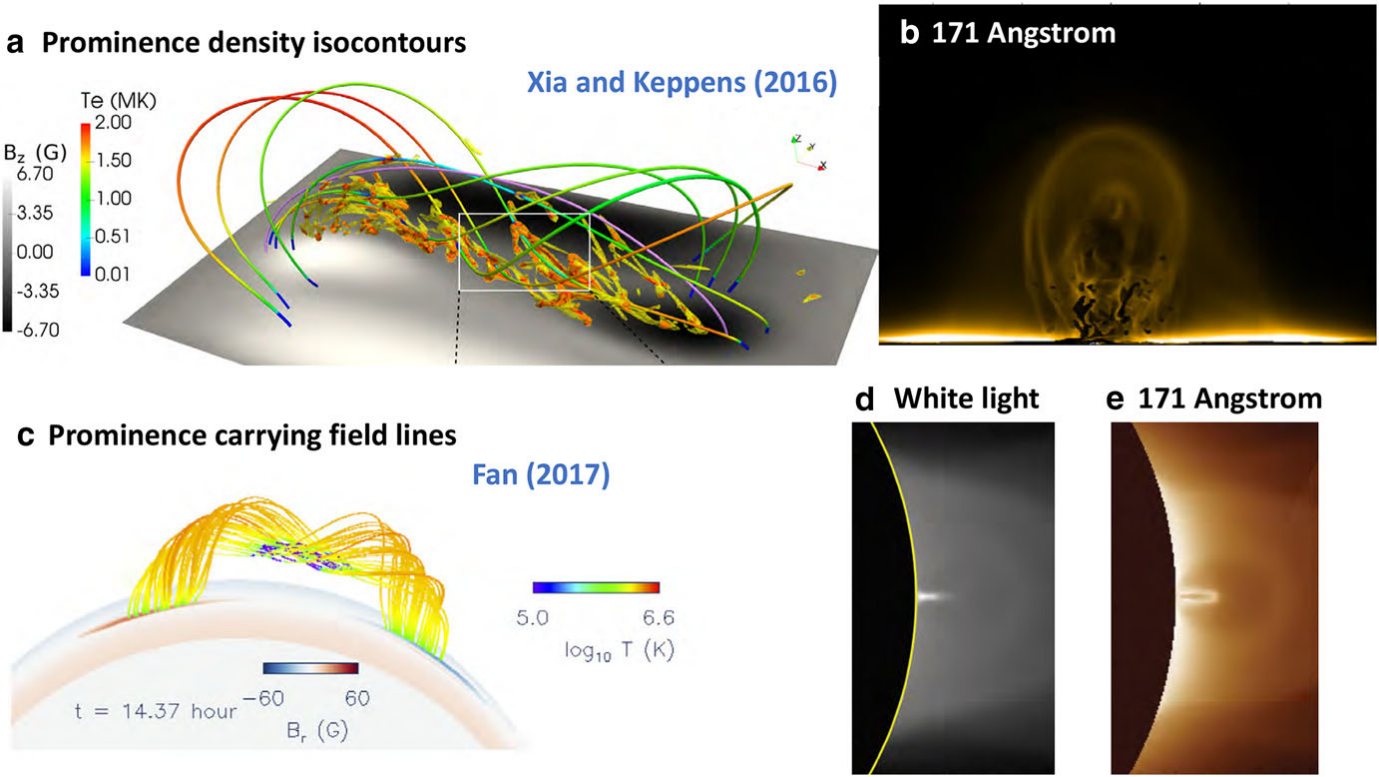
Simulations in which prominences dynamically form within 3D magnetic structures (left) **a** Xia and Keppens (2016) and **c** Fan (2017) sample magnetic field lines with cool, dense prominence plasma supported in dips; (right) Synthetic observations of prominence material in absorption surrounded by a coronal cavity from **b** Xia and Keppens (2016) and **d** a simulation similar to Fan (2017) that was presented in Gibson et al. (2016). Images reproduced with permission, **a**–**c** copyright by AAS

Xia and Keppens (2016) obtained their prominence in three steps. First, they constructed an isothermal flux rope in MHD equilibrium. Second, they added a hydrostatic vertically-structured atmosphere including a chromosphere, and solved the MHD equations with explicit treatment of the energy equation. Finally, they applied localized heating at the flux-rope foot points and condensed a prominence via TNE (Fig. 20a). The resulting prominence was fragmented and highly dynamic, with blobs and threads continuously forming in the dips of the flux rope and dragging the field lines downward to create vertical hedgerow-type structures. Prominence plasma was replenished through ongoing evaporation from the chromosphere and condensation upon the flux rope in the corona. This dynamic prominence was surrounded by a coronal cavity, with synthetic observations reproducing the relationship between cavity, prominence, and bright coronal flows within the cavity well (Fig. 20b).

Fan (2017) took a somewhat different approach, imposing a time-varying electric field at the lower boundary of a simulated quasi-steady coronal helmet streamer in a manner consistent with the kinematic emergence of magnetic twist. The MHD equations were solved including empirically-defined coronal heating, optically-thin radiative cooling and electron heat conduction. The result was a confined magnetic flux rope in quasi-equilibrium beneath the streamer, within which a cold, dense prominence formed due to a runaway radiative instability (Fig. 20c). As the prominence formed, its mass dragged the magnetic field downward, leading to a significantly non-force free magnetic configuration.

These two simulations differed in their set-up and in some of their objectives: Xia and Keppens (2016) provided a high-resolution view of prominence fine-structure dynamics within a coronal magnetic configuration, while Fan (2017) studied the formation and evolution of the prominence in spherical coordinates, capturing its ultimate eruption into a background solar wind. It is interesting therefore that the two simulations obtained such similar—and observationally realistic—prominence-cavity morphologies (Fig. 20b/d/e). They also demonstrated qualitatively similar dynamic processes controlling the prominence’s condensation and ongoing evolution, in a manner extending upon but ultimatley consistent with previous 1D TNE simulations. Finally, both simulations noted significant departures from force-freeness as gravity dragged the magnetic field downward, in what was, however, still a relatively low-$\beta$ plasma with mass accumulating in magnetic dips.

## Conclusions

Bearing in mind that our magnetic skeleton is neither completely rigid nor unchanging, let us consider what we have learned. If magnetic dips represent the bones of the prominence, then perhaps we may think of magnetic separatrices and flux surfaces as the joints and tissues connecting them. The challenge is then to arrange these structures in such a way that the dual requirements of consistency with observations and MHD force balance are satisfied. The topologies we have discussed—sheared arcade, flux rope, and spheromak—represent possible magnetic skeleton models that would achieve this. Based on the research reviewed here, we come to a few general conclusions:

1. Both the sheared arcade and flux rope models are consistent with the basic observations of the prominence spine, barbs, filament channel, and counterstreaming flows. A spheromak configuration, although consistent with the prominence spine and chirality properties, is not consistent with most linear-polarization observations of coronal cavities (as discussed in Sect. 3.3).
2. Some prominence-related observations strongly imply a flux rope model, including cavities with their nested toroidal structures and flows, well-defined (non-erupting) sigmoidal loops, and filaments/sigmoids that survive a partial eruption. It must be emphasized that such structures may exist in quasi-equilibrium for many hours or even days; however, ultimately they do tend to erupt. It is possible that there is a spectrum—from sheared arcade, to flux rope with BPSS, to flux rope with underlying X line—that may be directly associated with the stability of the prominence. It is also possible that the topological stage at which stability is lost differs for active region vs. quiescent prominences.
3. Many of the assumptions that underlie the construction of a magnetic skeleton, including the necessity of dips and the force-free nature of the prominence and surrounding corona, should be examined in light of the full thermodynamics of the prominence-corona environment. The assumptions may hold to first order, but, in particular when comparing models to observations, a deeper level of understanding is needed beyond the bones of the skeleton. Numerical simulations are of particular use here, as they provide a means of simultaneously treating the global magnetic configuration and the thermodynamic properties of the prominence.

A final, personal reflection: It is clear that by selecting particular observations one can find evidence for any or all of the models/ magnetic topologies discussed in this review. This brings to mind the story of the elephant and three blind men, each of whom had dramatically different perspectives on what they were encountering. Ultimately, any model that fails to explain *all* of the observations is suspect; this motivates the hyperrealism of MHD thermodynamic simulations. I expect that future efforts are likely to move away from idealized models and towards data-driven simulations, with better and more comprehensive observations a necessary requirement for progress.

Despite all of the caveats presented in this review, I feel that the concept of a (dynamic) magnetic skeleton will be useful to such endeavors. The skeleton is a framework within which observations of the prominence and its coronal environment may be boiled down to essentials, so that a data-driven, 3D MHD-thermodynamics approach to modeling the prominence in all its complexity will be possible.
